# Helium Incorporation into Scandium Fluoride, a Model Negative Thermal Expansion Material

**DOI:** 10.1021/acs.chemmater.4c03329

**Published:** 2025-02-04

**Authors:** Shangye Ma, Samuel J. Baxter, Changyong Park, Stella Chariton, Antonio M. dos Santos, Jamie J. Molaison, Angus P. Wilkinson

**Affiliations:** † School of Chemistry and Biochemistry, Georgia Institute of Technology, Atlanta, Georgia 30332-0400, United States; ‡ HPCAT, X-ray Science Division, Argonne National Laboratory, Argonne, Illinois 60439, United States; § Center for Advanced Radiation Sources, The University of Chicago, Argonne, Illinois 60637, United States; ∥ Neutron Scattering Division, Oak Ridge National Laboratory, Oak Ridge, Tennessee 37831, United States; ⊥ School of Materials Science and Engineering, Georgia Institute of Technology, Atlanta, Georgia 30332-0245, United States

## Abstract

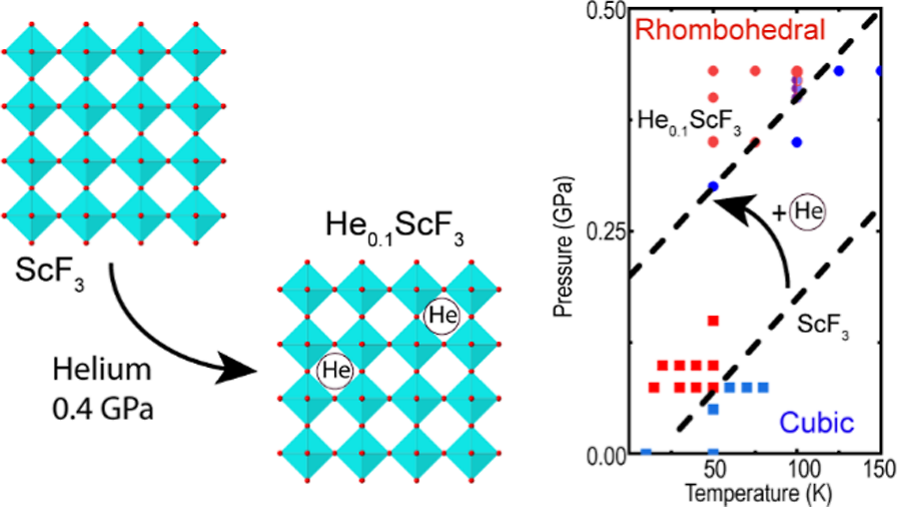

Scandium trifluoride is a model negative thermal expansion (NTE) material. Its simple structure can be described as an A-site vacant perovskite, and it shows isotropic NTE over a very wide temperature range (up to ∼1100 K), due to transverse vibrational motion of the fluoride. Like many framework NTE materials, it undergoes a phase transition at low pressures, adopting a rhombohedral (*R*3̅*c*) structure at >0.7 GPa and 300 K in commonly used nonpenetrating pressure media, such as silicone oil. High pressure X-ray diffraction data and gas uptake/release measurements indicate that, on compression in helium above ∼200 K, helium is inserted into ScF_3_ to form the defect perovskite He_*x*_ScF_3_. The incorporation of helium stiffens the structure and changes its phase behavior. At room temperature, complete filling of the structure with helium does not occur until >1.5 GPa. On compression, a cubic perovskite structure is maintained until ∼5 GPa. As the pressure was increased to ∼9.5 GPa, a further transition occurred at ∼7 GPa. The first transition at ∼5 GPa is likely to a tetragonal (*P*4/*mbm*) perovskite, but the detailed structure of the perovskite phase formed on compression above ∼7 GPa is unclear. Cooling down from 300 to 100 K in helium at ∼0.4 GPa leads to an approximate composition of He_0.1_ScF_3_. High pressure neutron diffraction measurements, in the temperature range 15–150 K show that the incorporation of helium increases the pressure at which the cubic (*Pm*3̅*m*) to rhombohedral (*R*3̅*c*) putative quantum structural phase transition occurs from close to 0 GPa to ∼0.2 GPa at 0 K.

## 1 Introduction

Scandium fluoride, ScF_3_, has attracted considerable attention since 2010, when it was first reported to display strong isotropic negative thermal expansion (NTE) over a wide temperature range.^1^ Much of this attention is likely attributable to its structural simplicity, when compared to other framework negative thermal expansion materials,^2,3^ making it an appealing model system to experimentally and computationally explore the details of negative thermal expansion driven by vibrational motion^4−7^ and several interesting phenomena such as marked pressure-induced softening^8−10^ and a quantum structural phase transition^11,12^ that accompany its NTE.

The NTE of framework solids, such as ScF_3_, is associated with vibrational modes (phonons) that have negative Grüneisen parameters. These are modes that move to lower frequency (soften) as a material’s volume is reduced at constant temperature.^13^ Framework NTE materials also often display structural phase transitions at modest pressures, leading to a loss of their NTE. This has led to many studies of NTE materials under pressure looking, for example, at phonons,^14−18^ phase transitions,^1,19^ and changes in both the coefficient of thermal expansion and bulk moduli on compression.^9,10,20−22^ In the case of ScF_3_, studies under pressure predate the discovery of its’ NTE in 2010. Early work included an exploration of polymorphism in ScF_3_, where an orthorhombic high-pressure form, ScF_3_-II, isostructural with β-YF_3_, was reported.^23,24^ Subsequently, there were ambient temperature experiments looking at the cubic (*Pm*3̅*m*) to rhombohedral (*R*3̅*c*) phase transition at ∼0.7 GPa, which is associated with *a*^–^*a*^–^*a*^–^ Glazer octahedral tilts^25−28^ that distort the ideal cubic structure of the parent phase. There have also been low-temperature high-pressure diffraction studies of ScF_3_ looking at its phase behavior^1^ and colossal pressure induced softening.^10^

Typically, in order to realize close-to-hydrostatic conditions, high pressure measurements are performed with a pressure transmitting fluid in the cell along with the sample. While many different media can be used, helium is particularly convenient for low-temperature high-pressure neutron scattering measurements when using a gas pressure cell, as it is more resistant to solidification on cooling and compression than alternative media. However, owing to its small size, under sufficient pressure, helium can penetrate some samples including quite dense solids such as cristobalite and silica glass,^29−31^ and modify their properties. Prior experimental work has shown that helium can be readily inserted, at modest pressure (<1 GPa), into the vacant A-sites of the cubic double ReO_3_-type NTE materials CaZrF_6_^32^ and CaNbF_6_,^33^ to form the perovskites [He_2–*x*_□_*x*_][CaZr]F_6_^34,35^ and [He_2–*x*_□_*x*_][CaNb]F_6,_^36^ where “□” denotes a vacancy on the perovskite A-site, and a computational study has suggested that this might be extended to other ReO_3_-type solids, including ScF_3_.^37^

The solid-state chemistry of helium is not extensive, but it is unusual and potentially of practical importance. In addition to inclusion compounds/clathrates, such as ice based materials,^38−40^ As_4_O_6_·2He^41^ and [He_2–*x*_□_*x*_][CaZr]F_6,_^34,35^ exotic compositions such as Na_2_He^42^ and HeK_2_O^43^ have been prepared^42^ or proposed based on computation.^43^ The incorporation and migration of helium in actinide oxides has been studied because of its relevance to the nuclear fuel cycle.^44−47^ Phases that incorporate helium have also attracted interest for their potential as traps, or sinks, for helium that might otherwise migrate to grain boundaries and lead to the failure of fusion reactor structural components.^48,49^

The current work was undertaken, primarily, to increase our understanding of solids that contain helium, but it should also serve as an additional note of caution for those conducting high pressure studies of materials using helium as a pressure transmitting medium. We report that helium can be quite readily incorporated into ScF_3_ at room temperature, and that only ∼10% incorporation at a pressure of 0.43 GPa, to form He_0.1_ScF_3_, significantly changes its properties. The metal fluorine bonds in ScF_3_ are close in length (2.01 Å) to the metal oxygen bonds in many transition metal oxides, and the “pores” in ScF_3,_ which are defined by rings of four corner sharing octahedra, are similar or smaller than those occurring in many oxides. This suggests that even for pressures of less than 1 GPa, studies of transition metal oxides containing empty sites, could be influenced by helium incorporation. Example structure types where penetration could be a problem, if there are empty sites, include various bronzes and pyrochlores.

## 2 Experimental Methods

### 2.1 Materials

ScF_3_ (STREM Chemicals, 99.9%) was used as received for all of the reported experiments.

### 2.2 High-Pressure X-ray Powder Diffraction Measurements at Elevated Temperature

A ∼4:1 mixture of ScF_3_ and CaF_2_ powder was loaded, along with ruby balls, into a symmetric diamond anvil cell (DAC), which was equipped with 600 μm cullet diamonds and a laser drilled (Ø 350 μm) preindented rhenium gasket. The cell was sealed under high pressure helium using the GSECARS gas loading facility^50^ and mounted in an evacuated enclosure. The DAC was heated by a resistive heater surrounding the cell body. A thermocouple mounted on a brass plug, which was inserted into the side hole of the symmetric DAC, was used to monitor the temperature of the DAC body. After allowing the system to thermally equilibrate for several minutes, the sample and DAC body temperature are close to one another. During the diffraction measurements, the pressure in the DAC was principally determined using the unit cell volume of CaF_2_ and its high temperature equation of state.^51^ Uncertainties in the pressure determination of ∼0.1 GPa are possible.^51,52^ Diffraction data were acquired using the HPCAT beamline, 16-BM-D, at Sector 16 of the Advanced Photon Source, Argonne National Laboratory, using a focused X-ray beam (∼3.5 × ∼4.7 μm fwhm) of wavelength 0.4133 Å (30.00 keV). The data were recorded on a MAR345 image plate detector with a sample to detector distance of ∼350 mm.

### 2.3 High-Pressure X-ray Powder Diffraction Measurements at Ambient-Temperature

A ∼3:2 ratio of powdered ScF_3_ and CaF_2_ was loaded, along with some ruby balls, into a BX90 diamond anvil cell, which was equipped with 600 μm cullet diamonds and a laser drilled preindented stainless-steel gasket. Powder diffraction data were recorded at pressures up to ∼10 GPa using the GSE-CARS beamline, 13-BMD, at Sector 13 of the Advanced Photon Source, Argonne National Laboratory. A focused beam (6 μm × 12 μm FHWM) with a wavelength of 0.3344 Å (37.00 keV) was employed along with a Pilatus 1 M CdTe detector.

### 2.4 High-Pressure Neutron Powder Diffraction Measurements at Low Temperature

∼0.6 g of ScF_3_ powder was loaded into an autofrettaged aluminum (alloy 7075) gas pressure cell, which is one of the standard gas cells available to SNS users,^53^ and has a working pressure limit of 0.48 GPa. A radial collimator with gadolinium blades was clamped to the outside of the pressure vessel to reduce the scattering from the pressure vessel body. The cell was affixed to a sample stick, and used in a closed cycle refrigerator. Helium pressure in the cell was controlled using a hand operated high-pressure syringe pump. Neutron powder diffraction data were collected on the instrument SNAP, at the Spallation Neutron Source, Oak Ridge National Laboratory. A 3 mm boron nitride collimator was used to define the beam at the sample. The instrument’s two detector banks were centered at 50° and 90° 2θ respectively.

### 2.5 Helium Uptake and Release Measurements

The reported results come from two sets of measurements using slightly different experimental arrangements. The following general procedure was employed for both sets of measurements.

A known mass of ScF_3_, contained in either the same design of sample cell that was used for the neutron diffraction study or a CuBe high pressure cell,^53^ was exposed to a target high-pressure of helium (between 0.30 and 0.45 GPa) at ∼300 K, and the sample was cooled to 100 K under constant high-pressure. After cooling to 100 K, some helium is effectively trapped inside the ScF_3_. The helium pressure was then vented from the system, and the system was evacuated and sealed. The ScF_3_ sample was warmed up from 100 K, in steps, to 300 K. As it was warmed up, the helium that had been trapped in the sample was able to migrate out of the ScF_3_ and produced a pressure increase (0.00–0.30 MPa) in the sample cell and the pipework connecting it to a pressure gauge. As the volume of this “head space” over the sample had previously been determined, from the pressure drop occurring as gas was allowed to expand from a known (calibrated) volume into it, the amount of helium released from the ScF_3_ could be calculated from the pressure increase in the “head space”.

### 2.6 Diffraction Data Analysis

Data from the high-pressure elevated temperature X-ray measurements (APS, HPCAT, 16BMD) were reduced, and fit, using GSAS-II.^54^ Data from the high-pressure ambient temperature X-ray measurements (APS, GSE-CARS, 13BMD) were processed using DIOPTAS^55^ and fit using GSAS-II.^54^ Data from the high-pressure neutron diffraction measurements (SNS, SNAP) were processed using MANTID and subjected to Rietveld analysis using GSAS-II.^54^

## 3 Results and Discussion

Initially, it was assumed that helium would not penetrate into ScF_3_ at 300 K due to the shorter Sc–Sc distance (4.01 Å),^1^ which can be considered as a measure of the pore aperture, when compared to the known metal–metal distances in CaZrF_6_ and CaNbF_6_ (4.24^32^ and 4.20 Å,^33^ respectively). Therefore, we first performed a high temperature (573 K) high-pressure XRD study and subsequently conducted a 300 K high-pressure XRD measurement as a control. However, the control experiment demonstrated that helium could penetrate into ScF_3_ at 300 K. Here, we report both results.

### 3.1 Elevated Temperature High-Pressure X-ray Measurements

**Figure 1:**
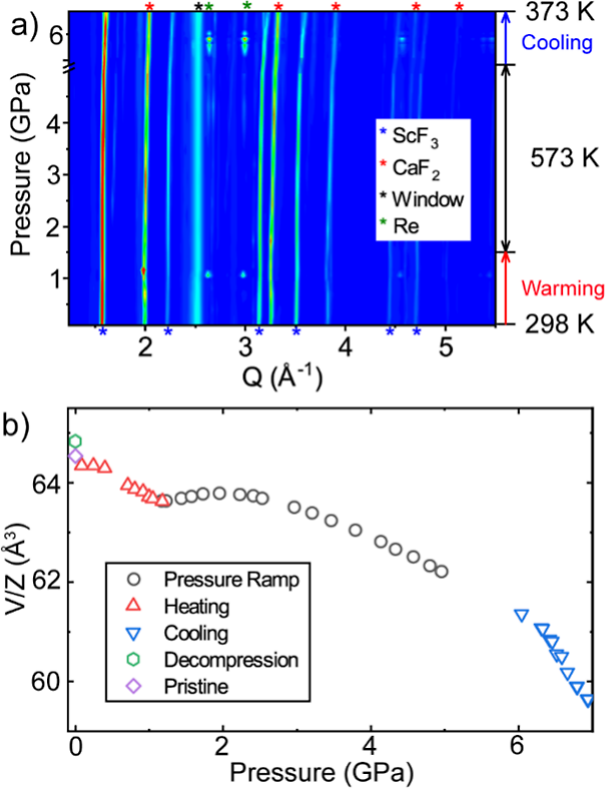
(a) X-ray diffraction data recorded on compression in helium during the high temperature diffraction experiment. The blue, red, green and black stars indicate the location of Bragg peaks from the ScF_3_, CaF_2_ pressure marker, rhenium gasket and scattering from the vacuum enclosure window material, respectively. (b) Unit cell volume per formula unit for ScF_3_ derived from the data shown in (a) along with values for pristine ScF_3_ (literature value at ambient pressure) and the decompressed and cooled sample. In the region where the sample is thermally well equilibrated (the pressure ramp at 573 K), the pressure uncertainties are likely similar to the symbol size.

The results presented in this section have previously been reported in the Ph.D. thesis of Dr. Baxter.^56^ Diffraction patterns from the measurements are shown in Figure 1 along with unit cell volumes for ScF_3_ determined by full profile fits, using a cubic ScF_3_ model. An example fit is shown in Figure S1. The gas loaded DAC, sealed at room temperature under ∼0.1 GPa helium, was heated in stages to 573 K (see Table S1), with diffraction data taken after each heating step. During the heating, the pressure in the DAC increased likely due to the softening of the gasket (Re) and spring washers (stainless steel), as well as the thermal expansion of the medium. We discuss the sample’s response only in the context of the He insertion and its influence on the extension of phase stability. After reaching 573 K and ∼1.2 GPa, data were collected at constant temperature as the pressure was increased in steps up to 4.95 GPa. After leaving the cell for 1.5 h at 573 K, it was cooled down in stages and further data were collected. On cooling, the pressure increased further, perhaps, due to the contraction of cell parts and restiffening of the spring washers. A similar increase in pressure on cooling has been observed by us in other measurements using this sample environment. The controlled cooldown was terminated at ∼373 K and 6.94 GPa. Data from the fully decompressed and cooled sample were collected later.

The diffraction data in Figure 1 show no indication of the cubic (*Pm*3*m*) to rhombohedral (*R*3̅*c*) phase transition typically seen at 0.7 GPa on compressing ScF_3_ at ambient temperature in a nonpenetrating medium, such as methanol–ethanol or silicone oil.^28^ This is consistent with a transition pressure of 1.4 GPa at 573 K, estimated by linear extrapolation of the reported cubic to rhombohedral phase boundary for ScF_3_^1^ with the assumption that no helium has been inserted into the structure. However, the unit cell volume increases during compression at 573 K over the pressure range 1.16–1.94 GPa, which clearly indicates helium insertion. Similar behavior has been reported on compressing CaZrF_6_ and CaNbF_6_ in helium at room temperature, and interpreted as a signature of helium penetrating into the structure and inflating the unit cell as more helium is incorporated. Conventional porous systems, such as some zeolites and MOFs, also show similar phenomena, along with changes in properties such as elastic stiffness, when compressed in fluids that can penetrate into their pore systems.^57,58^ It is notable that the maximum unit cell volume for ScF_3_ occurs at much higher pressure (∼1.9 GPa) than for CaZrF_6_ (0.8 GPa)^34^ and CaNbF_6_ (0.9 GPa).^36^ These differences could be due to the different pore sizes for these materials and/or the different measurement temperatures. This is discussed further in the next section. It is also notable that the bulk modulus estimated over the pressure range 3.2–5.0 GPa, *K* ∼94(2) GPa, (see Figure S2), is higher than that expected for cubic ScF_3_ without helium in the structure (∼60 GPa^59^). This is consistent with included helium stiffening the structure, through steric interactions with the framework fluoride. Prior high pressure experiments (<0.3 GPa) in silicone oil have shown that the bulk modulus of ScF_3_ is essentially invariant between 298 and 523 K, suggesting that the difference in bulk moduli between the current work in helium and prior studies in silicone oil is likely largely due to the insertion of helium in to the ScF_3_, and not temperature differences.^59^

### 3.2 Room Temperature High-Pressure X-ray Measurements

**Figure 2:**
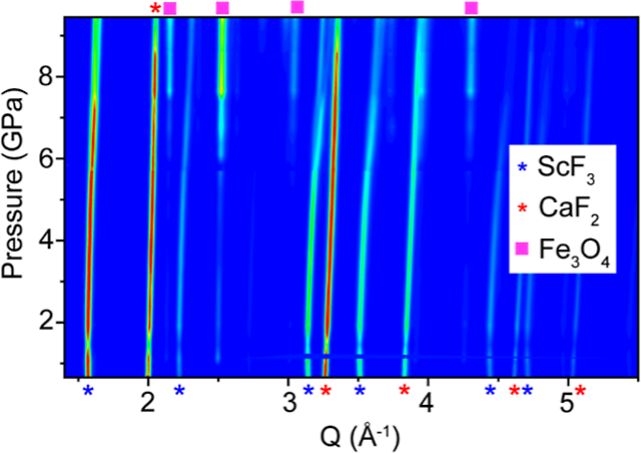
X-ray diffraction data recorded on compression in helium at 300 K. The blue and red stars indicate the location of Bragg peaks from the ScF_3_ sample and CaF_2_ pressure marker respectively, and the pink squares indicate the location of Fe_3_O_4_ peaks. The later was present due to oxidation of the stainless-steel gasket during laser drilling.

As the high temperature measurements demonstrated that helium is inserted into ScF_3_ on compression at 573 K, a high-pressure diffraction study was done at ∼300 K, to examine the possibility that helium can also access the interior of the structure at ambient temperature. High-pressure powder X-ray diffraction data, collected over the pressure range 0.64–9.45 GPa (43 patterns in total) at ∼300 K, are shown in Figure 2.

The diffraction data in Figure 2 do not show the well-known cubic (*Pm*3̅*m*) to rhombohedral (*R*3̅*c*) phase transition that is seen on compressing ScF_3_ to ∼0.7 GPa in nonpenetrating fluids, such as methanol–ethanol or anhydrous glycerin,^1,60−63^ and silicone oil.^28^ The absence of this transition at ∼0.7 GPa indicates that helium penetrates into ScF_3_ at room temperature to form [He_1–*x*_□_*x*_][ScF_3_] and modifies the phase behavior of the parent ScF_3_.

**Figure 3:**
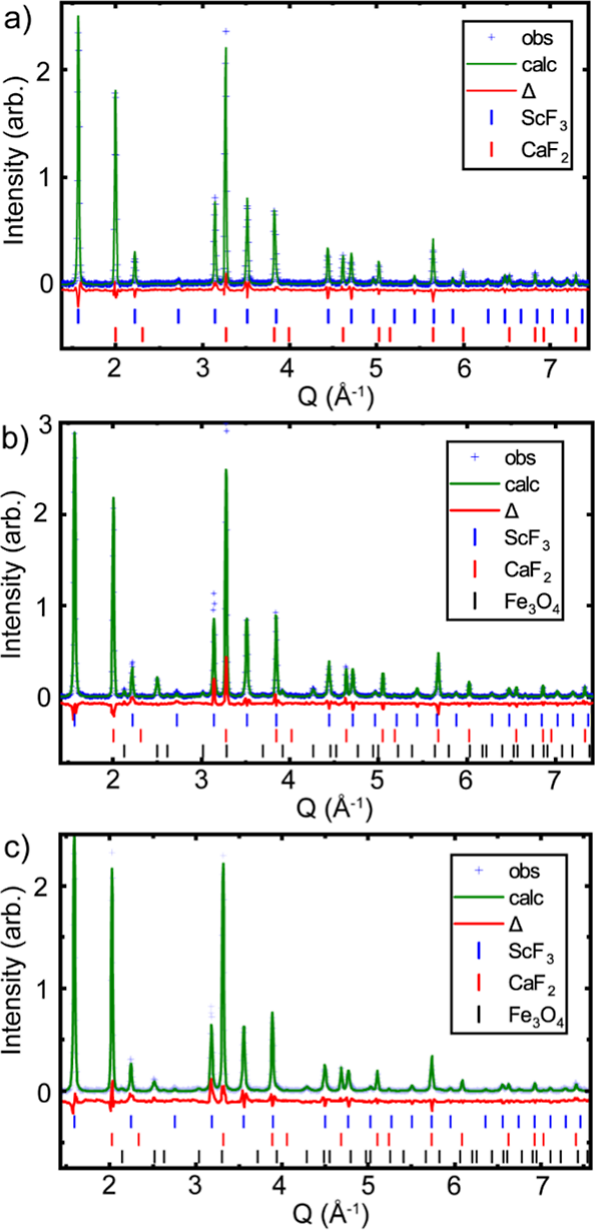
Rietveld fits to the 300 K high pressure diffraction data at different pressures using a cubic ScF_3_ model; (a) 0.72, (b) 1.93 and (c) 5.17 GPa. The blue, red and black tick marks indicate the predicated Bragg peak locations for ScF_3_, CaF_2_ and Fe_3_O_4_ respectively. *R*_F_ for CaF_2_ and ScF_3_ in the fits for panels (a–c) are (a) 11.7% and 21.5%, (b) 15.7% and 22.6%, and (c) 11.3% and 17.7%.

At the lowest pressures, the data can be fully accounted for with a model consisting of cubic ScF_3_ and the pressure marker CaF_2_ (see Figure 3a). At higher pressure, some extra peaks arise from Fe_3_O_4_, which is unfortunately present due the formation of oxidized material during the laser drilling of the stainless-steel gasket followed by brake away from the edges of the gasket hole on compression. The diffraction data are consistent with a model consisting of cubic ScF_3_, CaF_2_ and the spinel contaminant Fe_3_O_4_ at pressures up to and including 5.16 GPa (see the profile fits in Figure 3).

At higher pressures, the fit quality with a cubic ScF_3_ model deteriorates. Above 7.40 GPa, the first peak in the diffraction pattern (∼1.6 Å^–1^), which corresponds to the ScF_3_ cubic (100) peak, clearly splits (see Figure S3), indicating that the system is no longer cubic. An examination of this peak’s width as a function of pressure (Figure S5) suggests that there is an initial structural transition at ∼5 GPa. This is further supported by an examination of the cubic ScF_3_ (200) peak, which shows a shoulder developing above 5 GPa on the low-angle side of the peak and then, above ∼7.2 GPa, a redistribution of the intensity with the originally weaker component on the lower angle side of the split peak becoming stronger and the higher angle side becoming weaker (see Figure S3). These observations suggest that at least three different structural forms of [He_1–*x*_□_*x*_][ScF_3_] occur on compression at room temperature below 9.45 GPa; a cubic ReO_3_-type structure below ∼5 GPa, a second form between ∼5 and 7.2 GPa, and a third form above 7.2 GPa.

**Figure 4:**
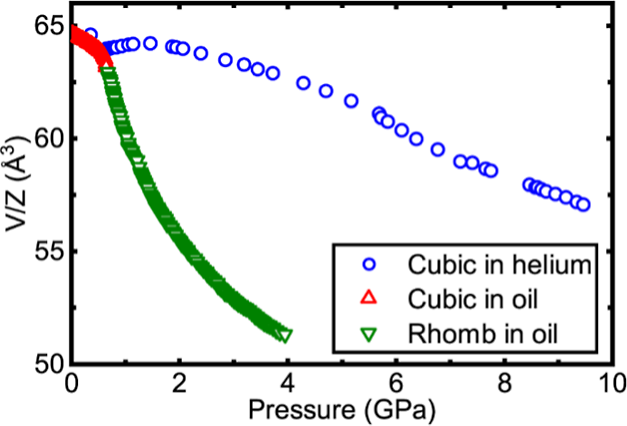
A comparison of the unit cell volume per formula unit for ScF_3_ compressed in a nonpenetrating low molecular weight silicone oil and helium. Red triangles, cubic ScF_3_ compressed in silicone oil; green triangles, rhombohedral ScF_3_ compressed in silicone oil and blue circles ScF_3_ compressed in helium. Note that a cubic model has been assumed for all data acquired using a helium pressure medium for simplicity and consistency in the comparison.

In Figure 4, the unit cell volume per formula (*V*/*Z*) unit for ScF_3_, obtained from Rietveld analyses assuming a cubic ReO_3_-type model at all pressures, is compared with that reported in a prior X-ray diffraction study using a silicone oil pressure medium.^28^ The assumption of a cubic model over the entire pressure range, despite the fact that some peak widths vary nonuniformly with pressure and that some peak splitting occurred for pressures above ∼5 GPa, was made for the purpose of comparison over the entire pressure range. The behavior in helium is very different from that seen in silicone oil. In the oil, there is a pronounced softening on compression at ∼0.7 GPa, which is associated with a cubic (*Pm*3̅*m*) to rhombohedral (*R*3̅*c*) phase transition. In the *R*3̅*c* structure, which has *a*^–^*a*^–^*a*^–^ Glazer octahedral tilts,^25^ changes in Sc–F–Sc bond angle on compression, associated with the tilts, provide a facile volume reduction mechanism. However, in helium, *V*/*Z* increases on compression between 0.69 and 1.46 GPa. As noted in the prior section, this is attributable to the insertion of helium into the empty A-sites of the ReO_3_-type structure to form [He_1–*x*_□_*x*_][ScF_3_], which “inflates” the unit cell. The maximum in *V*/*Z* on compression at 300 K occurs at lower pressure (∼1.5 GPa) than at 573 K (∼1.9 GPa), which presumably reflects a change in the thermodynamics for helium incorporation as a function of temperature; as the fugacity of a gas decrease on heating at constant pressure, a higher pressure is needed to achieve the same fugacity at higher temperature. The observed maximum values of *V*/*Z* at 300 and 573 K are 64.24(1) and 63.80(1) Å^3^ respectively. The smaller value of *V*/*Z* for the higher temperature could have contributions from a variety of factors including the higher pressure for the *V*/*Z* maximum at 573 K, differences in helium occupancy, and changes in elastic properties with temperature. An increase in *V*/*Z* on compression in helium at 300 K has also been observed for CaZrF_6_^34,35^ and CaNbF_6_,^36^ with the maximum in *V*/*Z* versus pressure at ∼0.8 GPa for [He_2–*x*_□_*x*_][CaZr]F_6_^34^ and ∼0.9 GPa for [He_2–*x*_□_*x*_][CaNb]F_6._^36^ The significant difference between the pressure of the *V*/*Z* maximum for ScF_3_ at 300 K (∼1.5 GPa) and those for both CaZrF_6_ and CaNbF_6_ likely reflects the smaller volume of the empty A-site in ScF_3_ when compared to the other materials. The shortest F–F distance across the A-sites in the parent fluorides at room temperature are 5.99, 5.94, and 5.68 Å respectively for CaZrF_6_, CaNbF_6_ and ScF_3_. Racioppi et al. estimated the ambient pressure A-site pore volumes in CaZrF_6_ and ScF_3_ to be 27.5 Å^3^ and ∼19 Å^3^ respectively, which are both significantly larger than the reported van der Waals volume for helium at ambient pressure (∼10 Å^3^).^37^

As seen for the 573 K measurements, the insertion of helium into ScF_3_ at 300 K, to form [He_1–*x*_□_*x*_][ScF_3_], stiffens the cubic structure. A straight line fit to volume versus pressure, over the range ∼2.4–5.2 GPa, led to an estimated bulk modulus of 84(3) GPa (see Figure S6). This is lower than the 94(2) GPa bulk modulus that was estimated for helium containing ScF_3_ at 573 K over a similar pressure range (see Section 3.1), which is contrary to the typical expectation of temperature-driven softening. However, because of the curvature in *V*/*Z* versus P, the estimates for the bulk moduli are also nonstandard (e.g., typical Birch–Murnaghan equation of state is not applicable) and vary with the pressure range chosen for the fits.

*V*/*Z* versus pressure, as shown in Figure 4, displays anomalies between 5 and 6 GPa and also at just over 7 GPa. These are consistent with our earlier suggestions, based on the fit quality to the XRD patterns, the variation in peak width with pressure, and the observation of peak splitting, that the sample undergoes structural phase transitions at around 5 and 7.2 GPa. In a 2023 computational study of helium insertion into ScF_3_ and related fluorides,^37^ Racioppi and co-workers suggested that, at 0 K, compression of stoichiometric HeScF_3_ to ∼1 GPa would lead to a transition from a cubic (*Pm*3̅*m*) perovskite to a tetragonal perovskite with space group *P*4/*mbm* (Glazer tilts a^0^a^0^c^+^), and that the tetragonal structure would be stable up to ∼3 GPa. Upon further compression, a rhombohedral *R*3̅*c* perovskite structure (Glazer tilts, *a*^–^*a*^–^*a*^–^) would be adopted, and at ∼6.5 GPa, a transition to a *Pnma* perovskite structure would occur. A clean comparison of these predictions with our data is not possible, as our experiments were performed at finite temperature. However, the current diffraction data were examined in the light of these predictions.

As the formation of an *R*3̅*c* structure, with *a*^–^*a*^–^*a*^–^ tilts, does not lead to splitting or broadening of the cubic (100) and (200) peaks, and these are observed (Figures S3 and S5), the formation of an *R*3̅*c* structure in the pressure range examined can be excluded.

**Figure 5:**
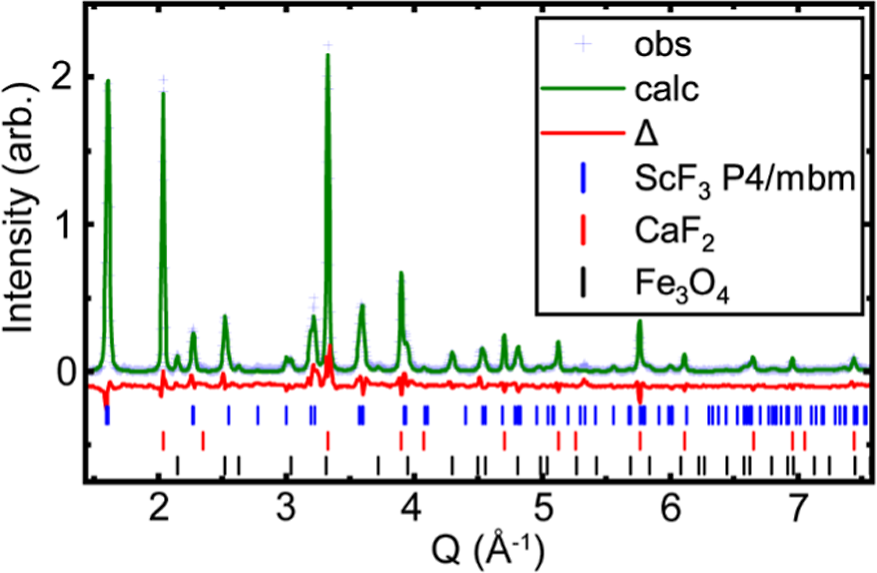
Rietveld fit to the 300 K X-ray diffraction data at 6.4 GPa using a *P*4/*mbm* model. The blue, red and black tick marks indicate the predicted Bragg peak locations for ScF_3_, CaF_2_ and Fe_3_O_4_ respectively. *R*_F_ for CaF_2_ and ScF_3_ respectively are 9.7% and 16.0%.

A transformation from cubic (*Pm*3̅*m*) HeScF_3_ to tetragonal (*P*4/*mbm*) leads to splitting of the original cubic (100) and (200) peaks into two components, with an intensity ratio of 2:1. The Glazer tilt system *a*^0^*a*^0^*c*^+^ should lead to *a*/*c* < √2, as the rotation of octahedra about the *c*-axis leads to a reduction of the a and *b* axes lengths, so the higher intensity component of the 2:1 split should be on the high angle side of the split peak. This is inconsistent with the splitting seen above ∼7.2 GPa, where the high intensity component is seen on the low angle side of the split (100) and (200) peaks. However, it is consistent with the splitting seen in the (200) peak below ∼7.2 GPa. A Rietveld refinement using a *P*4/*mbm* perovskite model and the 300 K, 6.4 GPa data gave a reasonable fit (Figure 5), although the fit quality and low resolution of the data, combined with poor sampling statistics, could not entirely preclude other possibilities.

The group theoretical analysis of octahedral tilting in perovskites by Howard and Stokes^26,27^ indicates that *P*4/*mbm* is a subgroup of *Pm*3̅*m*, and that a second order transition from *Pm*3̅*m* to *P*4/*mbm* symmetry via tilting of octahedra is possible. This analysis also shows that further second order transitions from *P*4/*mbm* symmetry to either *Immm* (*a*^+^*b*^+^*c*^+^), *Cmcm* (*a*^0^*b*^+^*c*^–^) or *Pnma* (*a*^+^*b*^–^*b*^–^) are possible. However, within the limited resolution of our data, a splitting of the cubic (100) or (200) peaks into the three components that these orthorhombic space groups imply is not seen. The splitting pattern of these peaks at high pressure is consistent with tetragonal symmetry, but with *c*/*a* < 1. This is not expected for a tetragonal system involving a single octahedral tilt but, interestingly, a similar situation was also observed when fitting a tetragonal model to a low temperature high pressure form of [He_2_][CaZr]_6_.^34^

### 3.3 Gas Uptake and Release

**Figure 6:**
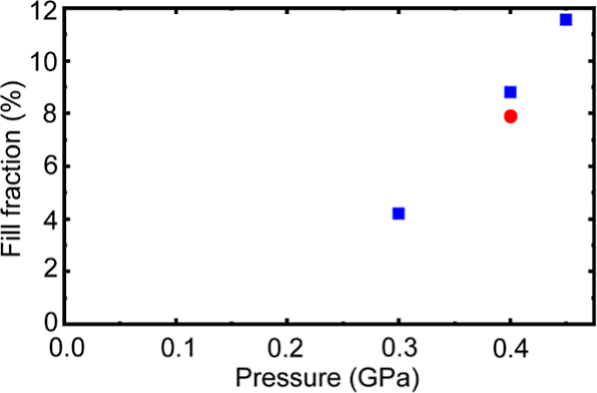
Fraction of A-sites occupied by helium in [He_1–*x*_□_*x*_]ScF_3_ after cooling the material to 100 K under different constant applied helium pressures. The results shown using red circles and blue squares were obtained using different experimental arrangements.

The fraction of A-sites occupied by helium, after cooling [He_1–*x*_□_*x*_]ScF_3_ to 100 K under different constant applied-helium pressures is shown in Figure 6. These values were estimated as described in the experimental section. The A-site occupancies are much lower than those previously observed for both [He_2–*x*_□_*x*_][CaZr]F_6_ and [He_2–*x*_□_*x*_][CaNb]F_6_ under similar conditions.^35,36^ In the case of [He_2–*x*_□_*x*_][CaZr]F_6,_ a fill fraction of ∼55% was achieved on cooling a sample to 100 K under 0.5 GPa helium,^35^ and for [He_2–*x*_□_*x*_][CaNb]F_6,_ a fill fraction of ∼60% was achieved on cooling the sample under 0.4 GPa helium.^36^ The kinetics for helium migration in and out of ScF_3_ are also different from those observed for CaZrF_6_ and CaNbF_6_. Gas uptake and release measurements for CaNbF_6_ indicated that helium diffusion out of the structure, on the time scale of the measurements, started at between 125 and 150 K, and for CaZrF_6_ the onset of helium diffusion occurred at ∼175 K. For ScF_3_, rapid migration of helium became apparent at above 200 K (see Figure S7). The lower observed fill fractions for ScF_3_, when compared to CaNbF_6_ and CaZrF_6_, likely arise from a combination of kinetic and thermodynamic factors. Thermodynamics come into play because the pore volume associated with the A-sites in ScF_3_ is less than that in both CaZrF_6_ and CaNbF_6_, so the helium fugacity needed to achieve a given equilibrium fractional occupancy at a specific temperature will likely be higher. The kinetics are important because the gas uptake values are not equilibrium measurements at a precisely defined temperature. The cool down to 100 K takes several hours due to the limited performance of the cryostat. While cooling under constant pressure, helium will exchange between the solid and the surrounding fluid until the kinetics slow down sufficiently that the system falls out of equilibrium at some “effective” equilibrium temperature. The temperature at which this occurs will be higher for ScF_3_ than for CaNbF_6_, due to the slower kinetics for ScF_3_. As the helium fugacity increases on cooling at constant gauge pressure, the higher effective equilibrium temperature for the ScF_3_ measurements implies a lower helium fugacity for the same gauge pressure than that for the experiments with CaNbF_6_.

As the pressure cell could not be cooled rapidly, due to the limited cooling power of the cryostat, and the kinetics for helium uptake and release slow down significantly on cooling from room temperature to 100 K, it is possible that, after cool down the perovskite, [He_1–*x*_□_*x*_]ScF_3_, contains a nonuniform distribution of helium. This could lead to individual grains of perovskite with a helium concentration gradient, where the surface region is richer in helium than the core. Such a concentration gradient would likely be associated with a distribution of lattice constants (microstrain) for the sample and some broadening of the phase transition pressure. We comment further on this possibility in the next section.

### 3.4 Neutron Diffraction

A high-pressure low-temperature powder neutron diffraction study was undertaken to examine the effect of helium insertion on the cubic-rhombohedral phase boundary at low temperature. This phase boundary, and its connection to the NTE of ScF_3_, has received prior attention in the context of a quantum structural phase transition at just above ambient pressure.^11,12,64,65^ The putative transition temperature at zero pressure was estimated to be −39 K by extrapolation of the temperature dependent frequency of the R point soft mode.^11^ This, along with the reported phase boundary P/T slope, was used to estimate a transition pressure of ∼750 bar (0.075 GPa) at 0 K.^11^

Two groups of neutron diffraction measurements were made. In the first group, data were taken as the sample was compressed to 0.43 GPa at 290 K, and then cooled to 50 K under pressure. Further data were then recorded as pressure–temperature space was explored at low temperature. In a second group of measurements, the sample was cooled with no applied helium pressure and then pressure–temperature space was explored. Based on the behavior observed during the 300 K X-ray experiments, and the gas uptake and release measurements, cooling under an applied pressure of 0.43 GPa helium leads to the formation of [He_1–*x*_□_*x*_]ScF_3_ with *x* ∼ 0.9, whereas cooling under no applied helium pressure enables the study of helium free material.

**Figure 7:**
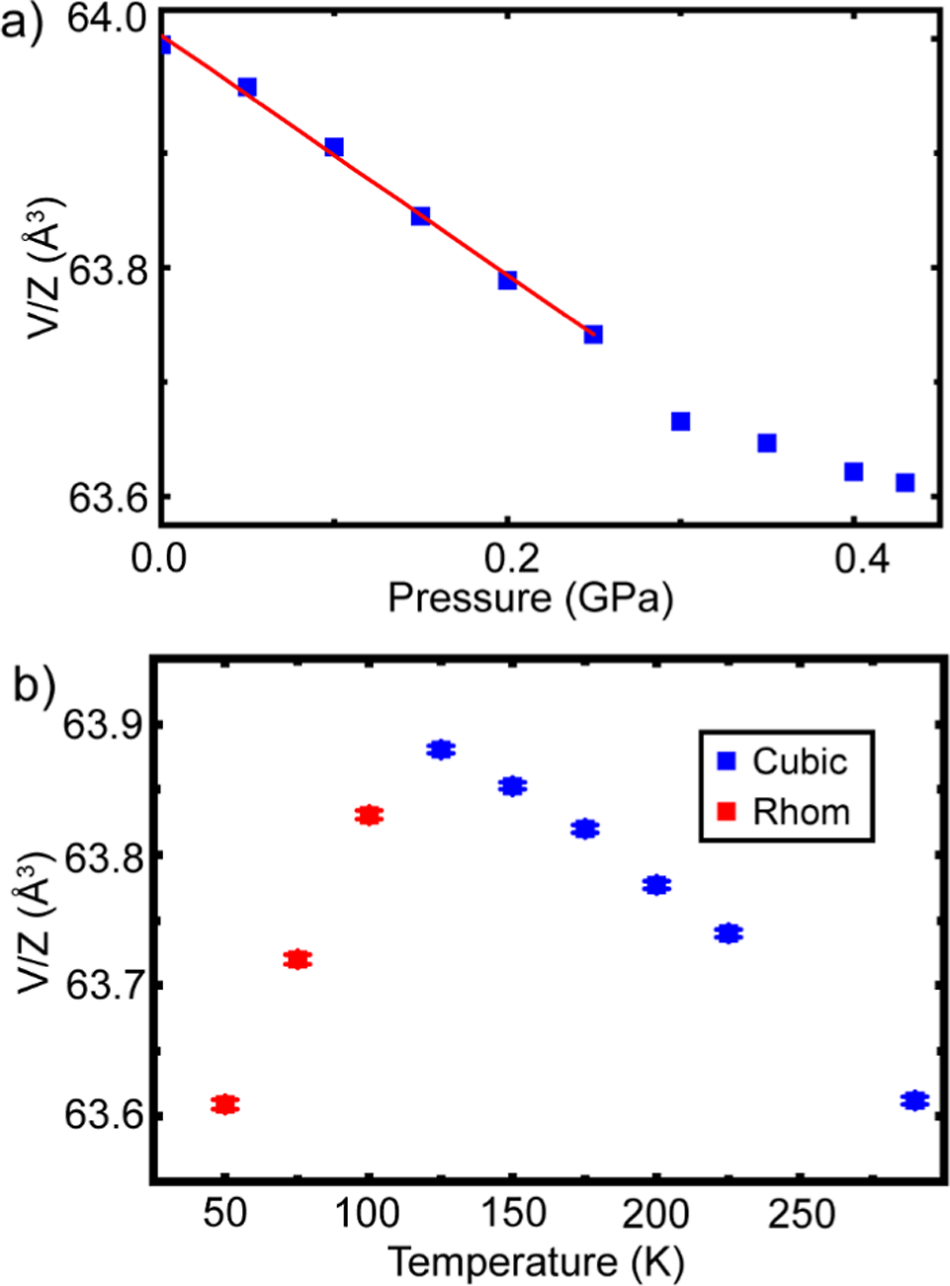
Unit cell volume per formula unit (a) during the initial compression of ScF_3_ in helium at 290 K and (b) during cooldown under 0.43 GPa helium pressure. The red line in (a) represents a straight-line fit used to estimate the bulk modulus for ScF_3_ in the low-pressure regime.

**Figure 8:**
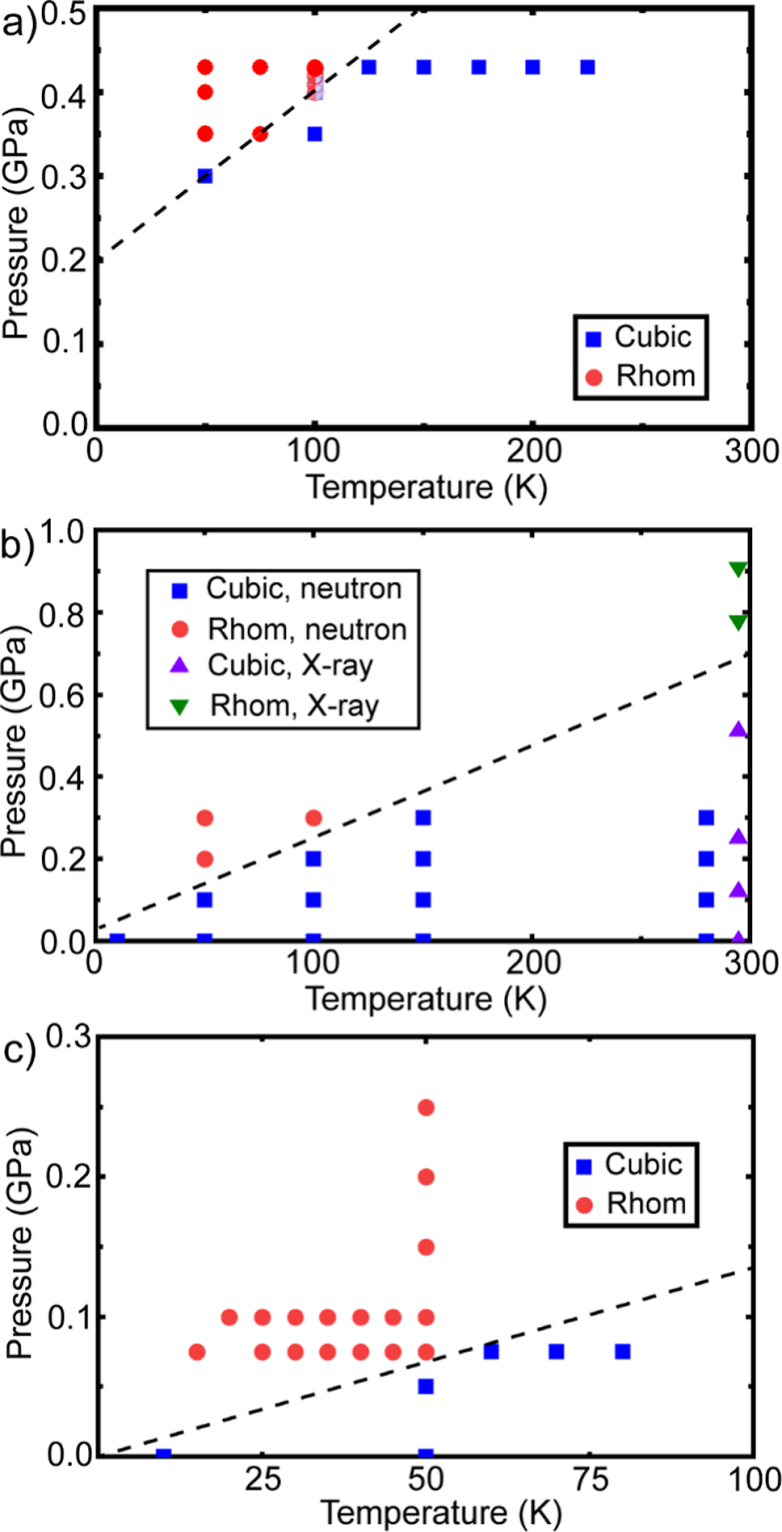
(a) Proposed low temperature phase diagram for He_0.1_ScF_3_. The pale blue symbols in this panel indicate points where the symmetry of the phase was ambiguous. (b) A redrawn version of the ScF_3_ phase diagram previously published by Greve et al.^1^ (c) A revised low temperature phase diagram for ScF_3_, based on the current data. Straight dashed lines have been placed to separate the cubic and rhombohedral regions.

Unit cell volume per formula unit, determined by Rietveld analyses, from the data recorded on compression at 290 K show a deviation from linearity above ∼0.25 GPa (Figure 7a), consistent with helium being incorporated into the structure above 0.25 GPa, and stiffening it, during this initial compression. Similar behavior was previously observed when compressing CaZrF_6_ and CaNbF_6_ in helium at room temperature.^35,36^ In the pressure range 0.0–0.25 GPa, a linear fit to unit cell volume versus pressure for the scandium fluoride sample gave a bulk modulus estimate of 61(2) GPa (see Figure 7), which is essentially identical to that for ScF_3_ at 295 K in nonpenetrating media, such as silicone oil. On subsequent cooldown under a constant 0.43 GPa helium pressure, negative thermal expansion was observed down to and including 125 K (Figure 7b), but the NTE appeared to decrease on cooling, which may be a precursor of the cubic to rhombohedral phase transition seen between 125 and 100 K. In the temperature range 100 to 50 K, positive thermal expansion was observed, which is typical for rhombohedral ReO_3_-type fluorides.^66−68^ Example Rietveld fits to data recorded at 225 K (cubic) and 50 K (rhombohedral) on cooldown under 0.43 GPa helium are shown in Figure S9. The observed transition temperature on cooling (125–100 K), as indicated by *V*/*Z* in Figure 7b and confirmed by the appearance of subtle superlattice peaks and peak splitting (see Figure S10), is much lower than one would expect for helium free ScF_3_ based on the previously published pressure temperature phase diagram (see Figure 8b), indicating that the incorporation of small amounts of helium (10% occupancy) has a significant stabilizing effect on the cubic phase and effectively raises the phase boundary. In Section 3.3, it was noted that on cool down from 300 K under high pressure helium, a helium concentration gradient could form in the grains of [He_1–*x*_□_*x*_]ScF_3,_ and that this would likely contribute to the development of microstrain induced peak broadening, as there is now a distribution of lattice constants associated with the distribution of compositions, on cooling. An examination of individual neutron diffraction peak widths, as a function of temperature, as the sample was cooled from 300 to 50 K under 0.43 GPa helium shows a modest continuous increase in peak widths on cooling (see Figure S11a). As might be expected from the increases in peak width, the refined microstrain parameter, from the Rietveld fits, also increases on cooling from 300 to 50 K (see Figure S11b). This could arise in part from a composition gradient within the sample grains.

To establish the cubic–rhombohedral phase boundary for He_0.1_ScF_3_ at low temperature, data were also collected as the pressure was varied at 50, 75, and 100 K (see Figure S12). An examination of the superlattice peaks associated with the cubic to rhombohedral transition leads to the proposed phase diagram in Figure 8a, where the phase boundary lies ∼0.2 GPa higher than that in the 2010 published phase diagram for the parent ScF_3_.^1^ The previously published phase diagram is redrawn in Figure 8b for comparison purposes. Further neutron diffraction measurements (Figures S13–S15) using the same sample of ScF_3_ that was used to generate the diagram in Figure 8a, but with cooling in the absence of high-pressure helium, indicate that the phase boundary for the parent ScF_3_ likely lies at lower pressure than indicated in Greve’s 2010 paper, and also lower than the 750 bar (0.075 GPa) at 0 K estimated as part of a low temperature X-ray inelastic scattering study of ScF_3_.^11^ The difference between the current work and the earlier study of Greve et al.^1^ is likely due to the different methods used to determine the phase boundary. In the work of Greve et al., the cubic and rhombohedral phases were distinguished by examining the splitting of the cubic (300), (221) peaks at ∼4.7 Å^–1^ (see Figure S12). However, based on the current measurements, the appearance of superlattice peaks at ∼2.6, 4.1, ∼6.0 and 7.5 Å^–1^ are a more sensitive indication of the transition to lower symmetry (see Figure S12), although there is still some uncertainty as to the transition temperature/pressure due to the limited counting statistics of the data.

## 4 Conclusions

Here we report on the formation of the defect perovskite, [He_1–*x*_□_*x*_]ScF_3,_ that results from the incorporation of helium into ScF_3_ at temperatures above 200 K under high pressures. The amount of helium incorporated into ScF_3_, for a given helium pressure, is less than that previously reported for the double ReO_3_-type materials CaZrF_6_ and CaNbF_6_, which is presumably due to the much smaller available volume of the empty A-sites in ScF_3_ when compared to the other materials. The maximum in *V*/*Z* for [He_1–*x*_□_*x*_]ScF_3_, as a function of helium pressure, occurs at a higher pressure than for both CaZrF_6_ and CaNbF_6_, and this maximum moves to higher pressure as the temperature is increased. The incorporation of helium into ScF_3_ stiffens the structure and changes its phase behavior. [He_1–*x*_□_*x*_]ScF_3_ shows two phase transitions below 10 GPa on compression at room temperature. Diffraction data for the first transition at ∼5 GPa is consistent with the formation of a *P*4/*mbm* perovskite structure, in agreement with the computational prediction of Recioppi and co-workers.^37^ However, the structure of the phase formed at ∼7 GPa remains unclear. Low temperature high pressure neutron diffraction measurements showed that the incorporation of small amounts of helium, to form He_0.1_ScF_3_, has a pronounced effect on the cubic to rhombohedral phase boundary at low temperatures, with the putative quantum structural phase transition (*Pm*3̅*m* to *R*3̅*c*) rising from less than 500 bar (0.05 GPa) to ∼2000 bar (0.2 GPa) at 0 K.

As the incorporation of helium into ScF_3_, CaZrF_6_^34,35^ and CaNbF_6_^36^ has been demonstrated to modify their elastic stiffness, (negative) thermal expansion, and phase behavior, the incorporation of helium, or other larger gaseous species such as molecular hydrogen or neon, could be viewed as a tool for tuning properties. However, the high pressures required for preparation and the limited stability of the gas included products limits the utility of this strategy.

## Supporting Information Available

Additional diffraction data profile fits, fits to determine bulk moduli, peak shape versus pressure at 300 K, peak width versus pressure at 300 K, gas release versus temperature, neutron diffraction data as a function of pressure and temperature. Tables of unit cell volume/constant versus pressure and temperature (PDF)
