# Supplementary material for: Helium Incorporation into Scandium Fluoride, a Model Negative Thermal Expansion Material
Source: Chem Mater. 2025 Feb 4;37(4):1657–66. doi: 10.1021/acs.chemmater.4c03329 (PMC11866548; doi:10.1021/acs.chemmater.4c03329)
Supplement: Supplementary file 1 — cm4c03329_si_001.pdf [file cm4c03329_si_001.pdf]

Supplementary Information for "Helium Incorporation into Scandium Fluoride, a model Negative Thermal Expansion Material"

Shangye Ma,[†] Samuel J. Baxter,[†] Changyong Park,[¶] Stella Chariton,[#] Antonio M. dos Santos,[‡] Jamie J. Molaison[‡] and Angus P. Wilkinson[†,§]*

[†]School of Chemistry and Biochemistry, Georgia Institute of Technology, Atlanta, GA 30332-0400, USA

[¶]HPCAT, X-ray Science Division, Argonne National Laboratory, Argonne, Illinois 60439, USA

[#]Center for Advanced Radiation Sources, The University of Chicago, IL 60637, USA.

[‡]Neutron Scattering Division, Oak Ridge National Laboratory, Oak Ridge, TN 37831, USA

[§]School of Materials Science and Engineering, Georgia Institute of Technology, Atlanta, GA 30332-0245, USA

Contents

Supplementary Figures

Figure S1. Rietveld fit to data for ScF_3 in helium at ~0.1 GPa and ~300 K, prior to heating to 573 K. Mylar was used as the widow material on the vacuum enclosure for the heated DAC, so that ruby fluorescence measurements could be made.

Figure S2. The bulk modulus of $HeScF_3$, at 573 K (300 ºC), in the pressure range ~3.2 – 5.0 GPa was estimated using a linear fit to (V/Z) versus pressure.

Figure S3. The behavior of a) the cubic (100) peak and b) the cubic (200) peak (~3.2 $Å^{-1}$) for ScF_3 on compression at 300 K in a helium containing DAC. The peak at just over 3.3 $Å^{-1}$ is from the CaF_2 pressure marker.

Figure S4. Diffraction data for ScF_3 on compression at 300 K in a helium containing DAC. Below 5.16 GPa, the sample is likely cubic. Not all of the reported measurements were from the same location on the sample.

Figure S5. Full width at half max versus pressure for the ScF_3 cubic (100) and CaF_2 (111) peaks. The ScF_3 (100) peak starts to broaden significantly above 5 GPa, suggesting a structural phase transition.

Figure S6. The bulk modulus of $HeScF_3$ in the pressure range ~2 – 5 GPa was estimated using a linear fit to (V/Z) versus pressure.

Figure S7. Helium released from a sample of ScF_3 that had been cooled to 100 K under a constant 0.4 GPa helium pressure. The blue curve was computed assuming a head space temperature of 300 K. This measurement corresponds to the red symbol on Figure 6 in the paper.

Figure S8. A linear fit to unit cell volume versus pressure was used to estimate the bulk modulus of ScF3 as it was compressed at 295 K in high pressure helium.

Figure S9. Rietveld fits to the neutron diffraction data recorded on cooldown under a 0.43 GPa helium pressure at a) 225 K (cubic) and b) 50 K (rhombohedral). These data have a small scattering contribution from the aluminum pressure cell body.

Figure S10. Neutron diffraction data for ScF_3 as a function of temperature on cooldown under 0.43 GPa helium. The black and red asterisks indicate respectively the locations of superlattice peaks and a peak shoulder/splitting associated with the cubic to rhombohedral phase transition. One component of the split peak seen at ~2.7 $Å^{-1}$ is due to scattering from the aluminum pressure cell body, which has not been completely blocked by the radial collimator.

Figure S11. a) Peak widths, for two peaks that do not split on going through the cubic to rhombohedral phase transition, and b) microstrain broadening parameter, from the neutron diffraction data that was recorded on cooling ScF_3 from 300 to 50 K under 0.43 GPa helium.

Figure S12. a) – c) Neutron diffraction data for ScF_3 that had been cooled down under 0.43 GPa helium leading to an approximate sample composition of $He_{0.1}ScF_3$. a) Patterns recorded at 50 K, b) 75 K and c) 100 K as the pressure was changed. d) V/Z obtained from Rietveld analyses of the diffraction data shown in a). e) V/Z obtained from Rietveld analyses of the diffraction data shown in c).

Figure S13. a) – e) Neutron diffraction data as a function of pressure at 50 K for ScF_3. a) presents an overview of the data collected. The red arrows indicate the location of superlattice peaks associated with rhombohedral ScF_3 and the red inverted "cup" indicates the location of a peak that splits in a notable fashion during the cubic to rhombohedral transition. b) – d) Enlargements of regions where there are superlattice peaks and e) a notable peak splitting. f) V/Z obtained from Rietveld analyses of the diffraction data. The significant softening seen between 0.05 and 0.15 GPa is consistent with a cubic to rhombohedral transition in this pressure range. Based on these data it is unclear if the sample was cubic or rhombohedral at 0.1 GPa. As the sample had been cooled down with no applied helium pressure, it did not contain helium.

Figure S14. a) – d) Neutron diffraction data for ScF_3 as a function of temperature at affixed pressure of 750 bar. a) An overview of all the data. b) – d) Regions where there are superlattice peaks from the rhombohedral phase. e) V/Z obtained by Rietveld analysis. The data underlying the values as open symbols were collected on cooling from 50 to 25 K and then there was a ~24 hour time gap, due to technical issues, before the data underlying the closed symbols were collected on warming from 15 to 80 K. During the time gap the sample position was apparently disturbed, leading to a small jump in lattice constants between the open and closed symbols. The positive thermal expansion associated with the open symbols suggests that the transition pressure lies above 50 K. The negative thermal expansion seen for the closed symbols between 40 and 60 K suggests a transition temperature below 60 K. As the sample was cooled down with no applied helium pressure, there should be no included helium in the material.

Figure S15. a) Neutron diffraction data as a function of temperature at 1000 bar for ScF_3. b) V/Z obtained by Rietveld analysis. The data in panel a) and the positive thermal expansion shown in panel b) are consistent with a rhombohedral to cubic transition temperature above 50 K at 1000 bar. As sample had been cooled down with no applied helium pressure, there should be no included helium in the material.

Supplementary Tables

Table S1. Temperatures, pressures, lattice constants, and cell volumes for ScF_3 (sample) and a CaF_2 pressure standard as determined by Rietveld analysis of the synchrotron X-ray diffraction data from the high temperature DAC experiment on HPCAT.

Table S2. Unit cell volume per formula unit versus pressure from a Rietveld analysis of the 300 K high-pressure X-ray powder diffraction data assuming that the sample is cubic at all pressures. It is likely not cubic above 5.16 GPa. The underlying data were collected on 13BMD at GSECARS.

Figures

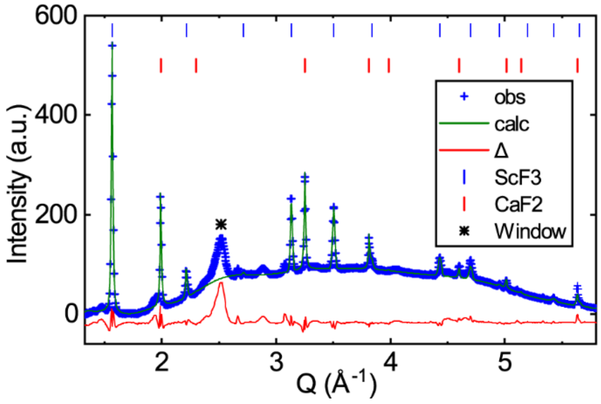

Figure S1. Rietveld fit to data for ScF_3 in helium at ~0.1 GPa and ~300 K, prior to heating to 573 K. Mylar was used as the widow material on the vacuum enclosure for the heated DAC, so that Ruby fluorescence measurements could be made.

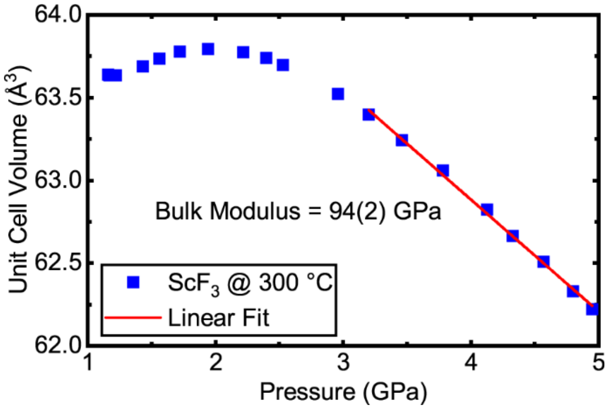

Figure S2. The bulk modulus of $HeScF_3$, at 573 K (300 ºC), in the pressure range ~3.2 – 5.0 GPa was estimated using a linear fit to (V/Z) versus pressure.

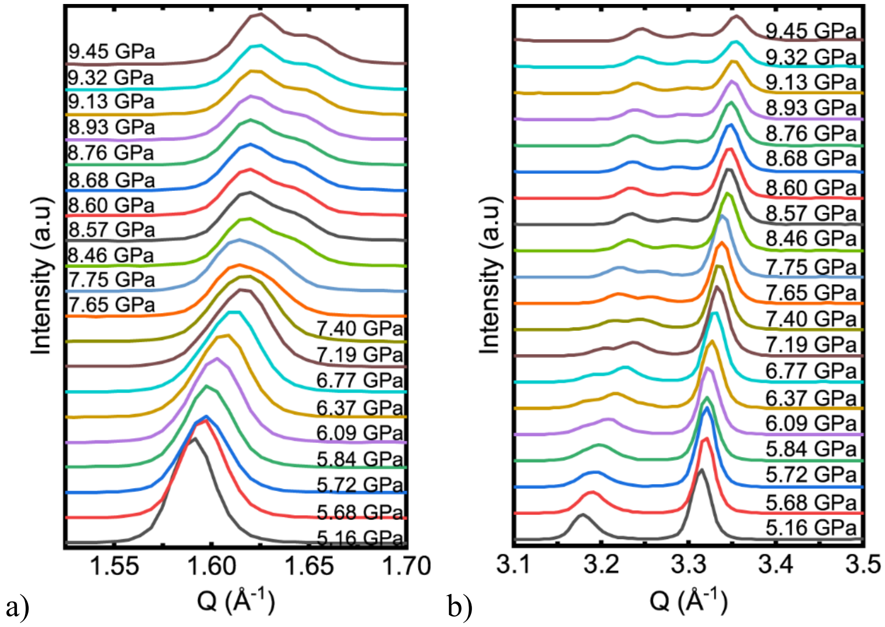

Figure S3. The behavior of a) the cubic (100) peak and b) the cubic (200) peak (~3.2 Å^{-1}) for ScF_3 on compression at 300 K in a helium containing DAC. The peak at just over 3.3 Å^{-1} is from the CaF_2 pressure marker.

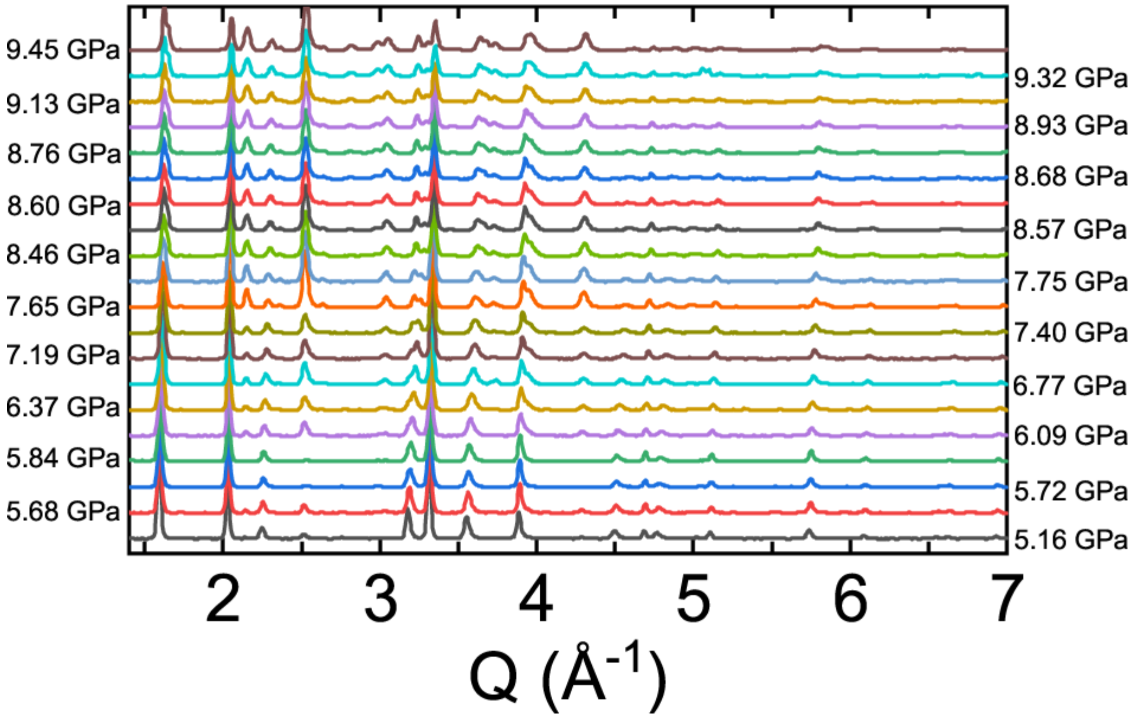

Figure S4. Diffraction data for ScF_3 on compression at 300 K in a helium containing DAC. Below 5.16 GPa, the sample is likely cubic. Not all of the reported measurements were from the same location on the sample.

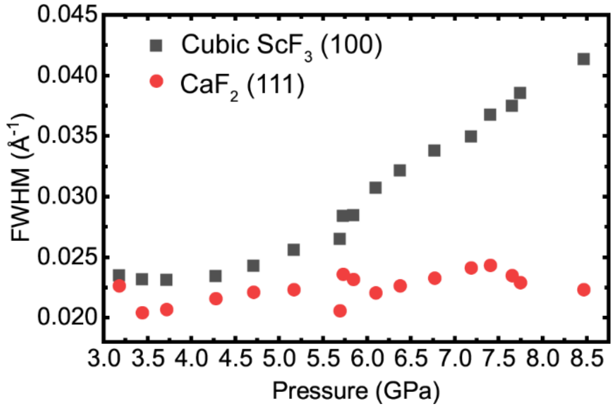

Figure S5. Full width at half max versus pressure for the ScF_3 cubic (100) and CaF_2 (111) peaks. The ScF_3 (100) peak starts to broaden significantly above 5 GPa, suggesting a structural phase transition.

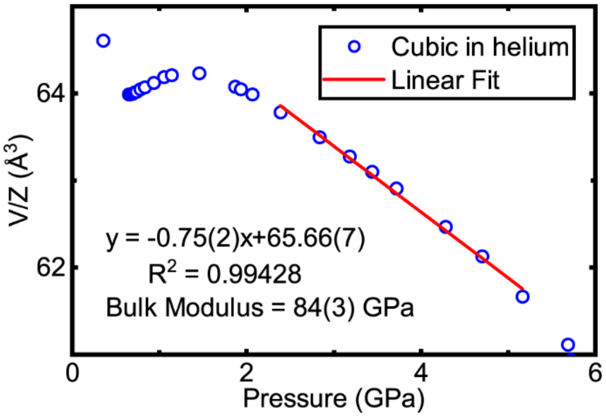

Figure S6. The bulk modulus of $HeScF_3$ in the pressure range ~2.4 – 5.2 GPa was estimated using a linear fit to (V/Z) versus pressure.

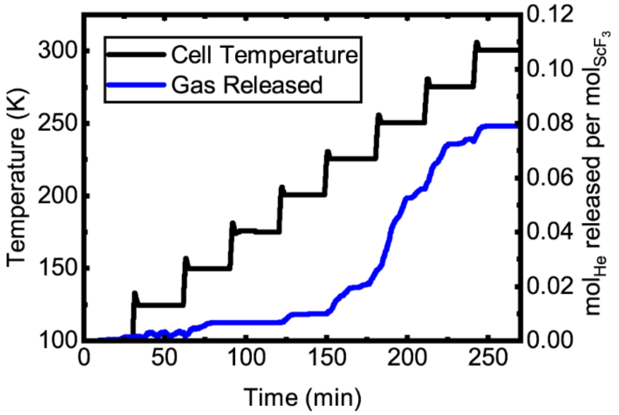

Figure S7 Helium released from a sample of ScF_3 that had been cooled to 100 K under a constant 0.4 GPa helium pressure. The blue curve was computed assuming a head space temperature of 300 K. This measurement corresponds to the red symbol on Figure 6 in the paper.

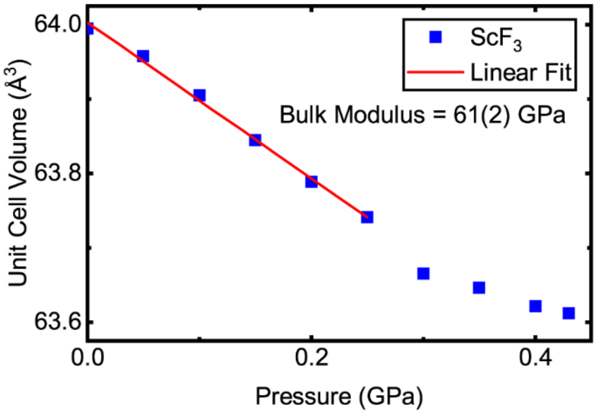

Figure S8. A linear fit to unit cell volume versus pressure was used to estimate the bulk modulus of ScF3 as it was compressed at 295 K in high pressure helium.

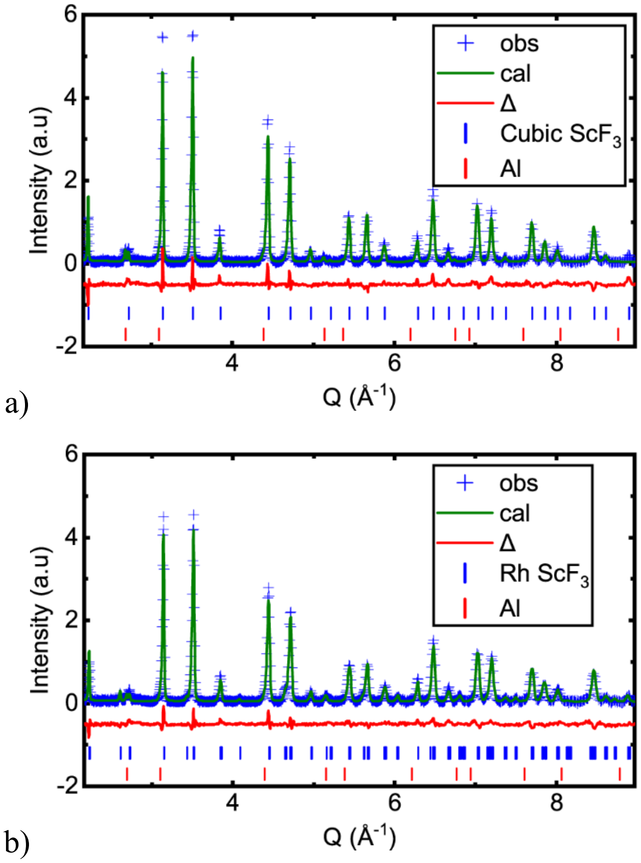

Figure S9. Rietveld fits to the neutron diffraction data recorded on cooldown under a 0.43 GPa helium pressure at a) 225 K (cubic) and b) 50 K (rhombohedral). These data have a small scattering contribution from the aluminum pressure cell body.

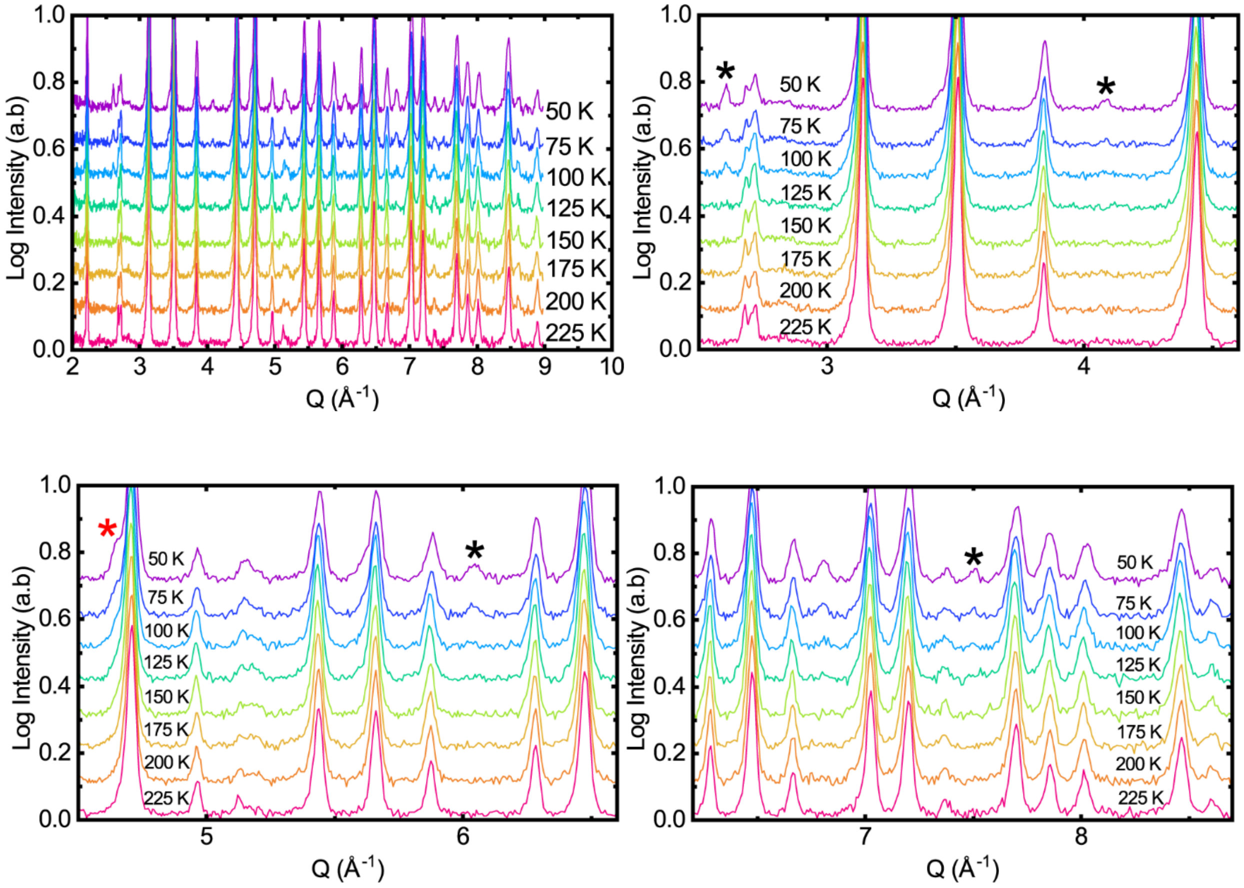

Figure S10. Neutron diffraction data for ScF_3 as a function of temperature on cooldown under 0.43 GPa helium. The black and red asterisks indicate respectively the locations of superlattice peaks and a peak shoulder/splitting associated with the cubic to rhombohedral phase transition. One component of the split peak seen at ~2.7 $Å^{-1}$ is due to scattering from the aluminum pressure cell body, which has not been completely blocked by the radial collimator.

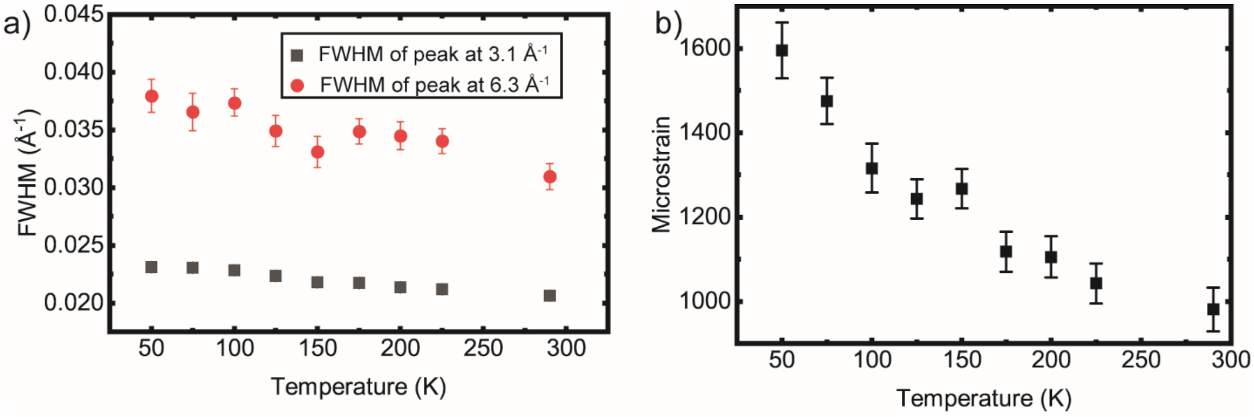

Figure S11. a) Peak widths, for two peaks that do not split on going through the cubic to rhombohedral phase transition, and b) microstrain broadening parameter, from the neutron diffraction data that was recorded on cooling ScF_3 from 300 to 50 K under 0.43 GPa helium.

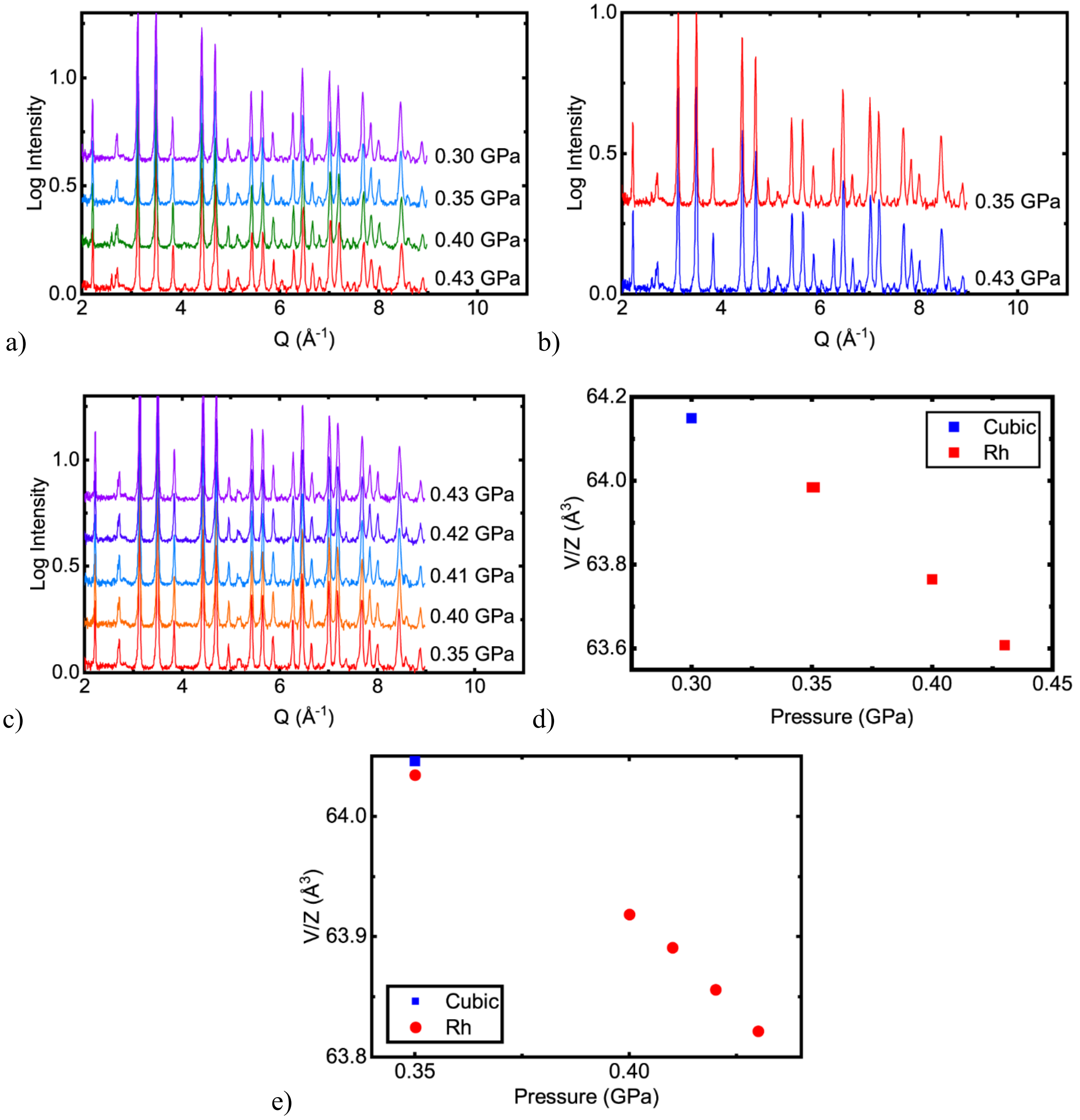

Figure S12. a) – c) Neutron diffraction data for ScF_3 that had been cooled down under 0.43 GPa helium leading to an approximate sample composition of $He_{0.1}ScF_3$. a) Patterns recorded at 50 K, b) 75 K and c) 100 K as the pressure was changed. d) V/Z obtained from Rietveld analyses of the diffraction data shown in a). e) V/Z obtained from Rietveld analyses of the diffraction data shown in c).

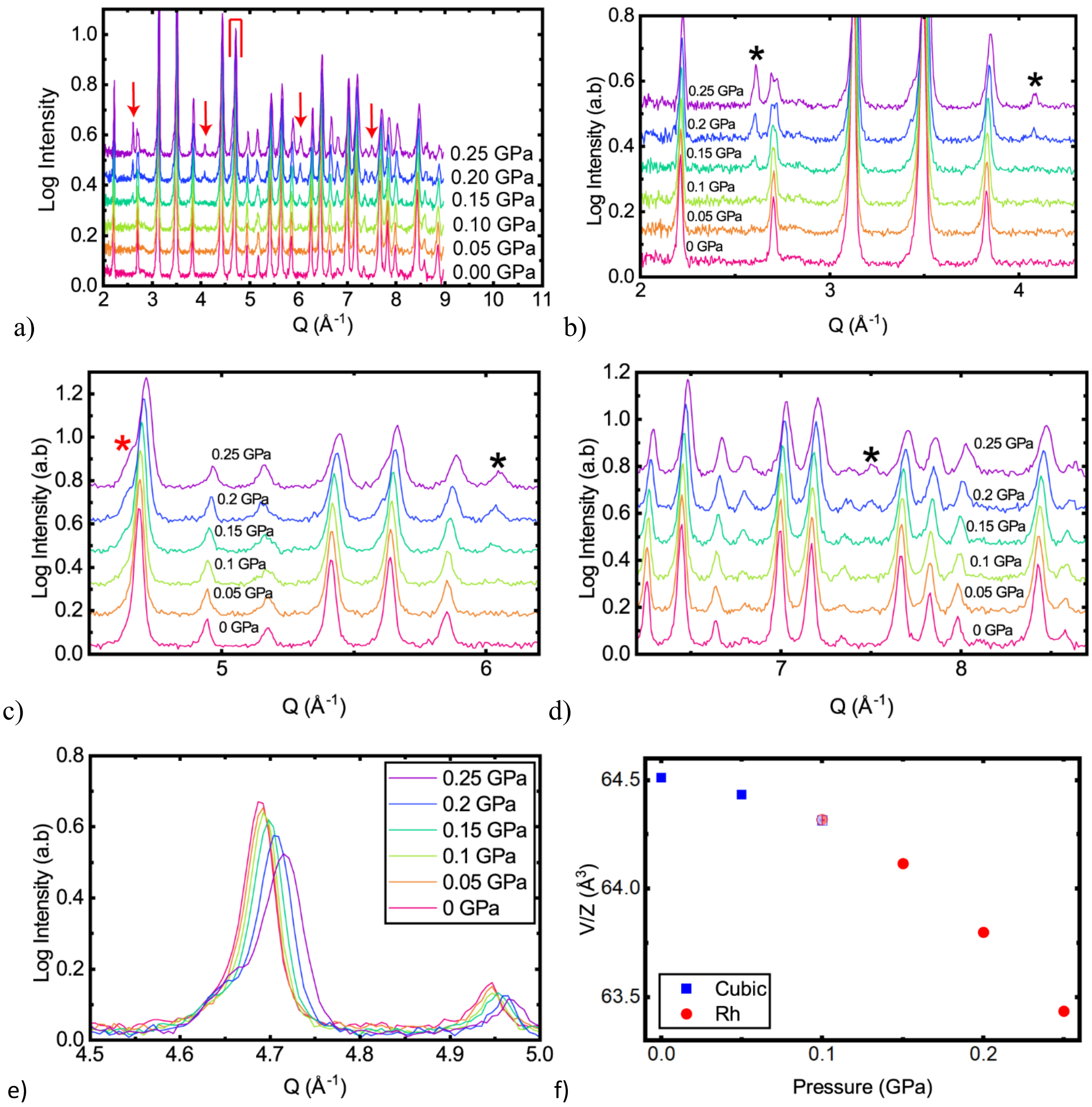

Figure S13. a) – e) Neutron diffraction data as a function of pressure at 50 K for ScF_3. a) presents an overview of the data collected. The red arrows indicate the location of superlattice peaks associated with rhombohedral ScF_3 and the red inverted "cup" indicates the location of a peak that splits in a notable fashion during the cubic to rhombohedral transition. b) – d) Enlargements of regions where there are superlattice peaks and e) a notable peak splitting. f) V/Z obtained from Rietveld analyses of the diffraction data. The significant softening seen between 0.05 and 0.15 GPa is consistent with a cubic to rhombohedral transition in this pressure range. Based on these data it is unclear if the sample was cubic or rhombohedral at 0.1 GPa. As the sample had been cooled down with no applied helium pressure, it did not contain helium.

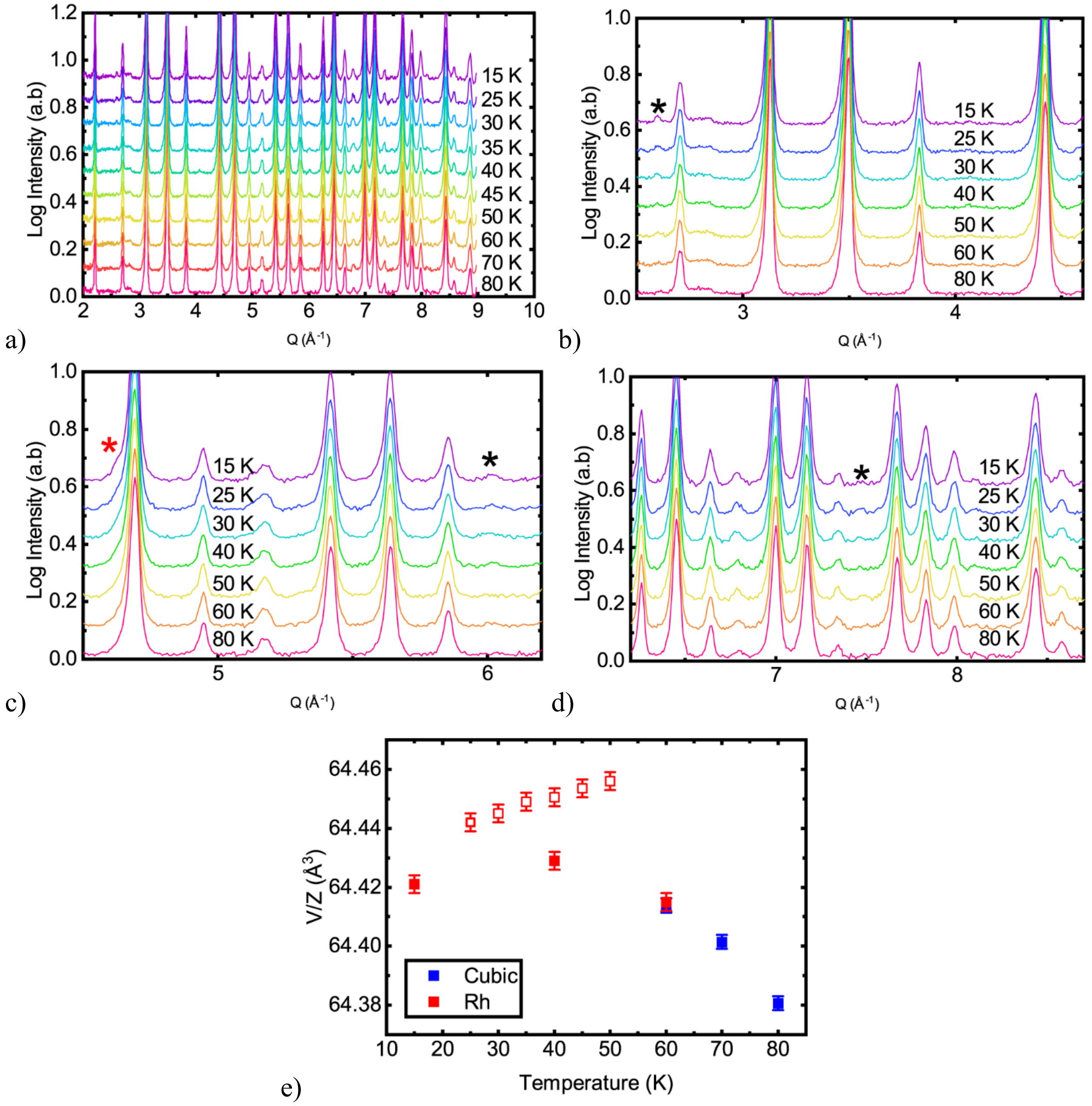

Figure S14. a) – d) Neutron diffraction data for ScF_3 as a function of temperature at affixed pressure of 750 bar. a) An overview of all the data. b) – d) Regions where there are superlattice peaks from the rhombohedral phase. e) V/Z obtained by Rietveld analysis. The data underlying the values as open symbols were collected on cooling from 50 to 25 K and then there was a ~24 hour time gap, due to technical issues, before the data underlying the closed symbols were collected on warming from 15 to 80 K. During the time gap the sample position was apparently disturbed, leading to a small jump in lattice constants between the open and closed symbols. The positive thermal expansion associated with the open symbols suggests that the transition pressure lies above 50 K. The negative thermal expansion seen for the closed symbols between 40 and 60 K suggests a transition temperature below 60 K. As the sample was cooled down with no applied helium pressure, there should be no included helium in the material.

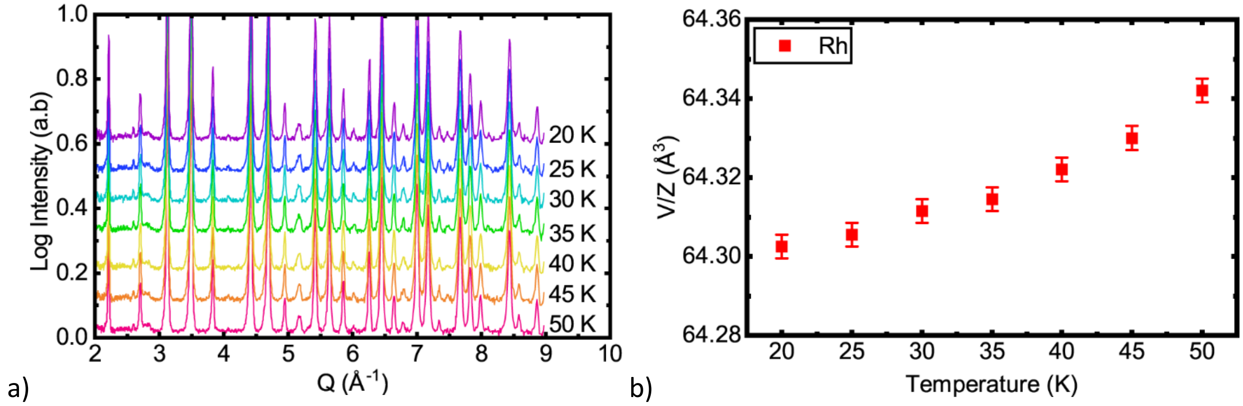

Figure S15. a) Neutron diffraction data as a function of temperature at 1000 bar for ScF_3. b) V/Z obtained by Rietveld analysis. The data in panel a) and the positive thermal expansion shown in panel b) are consistent with a rhombohedral to cubic transition temperature above 50 K at 1000 bar. As sample had been cooled down with no applied helium pressure, there should be no included helium in the material.

Tables

Table S1. Temperatures, pressures, lattice constants, and cell volumes for ScF_3 (sample) and a CaF_2 pressure standard as determined by Rietveld analysis of the synchrotron X-ray diffraction data from the high temperature DAC experiment on HPCAT.

Temperature (K)	**Pressure (GPa)**	**Rwp (%)**	**Sample Lattice Constant, a (Å)**	**Sample Volume, (Å^3)**	**CaF_2 Volume, (Å^3)**
298	0.09	2.7	4.0071(3)	64.34(2)	162.87(4)
298	0.25	3.1	4.0072(4)	64.35(2)	162.55(5)
323	0.40	3.2	4.0060(4)	64.29(2)	162.48(5)
373	0.71	2.7	3.9990(3)	63.95(1)	162.35(3)
425	0.81	2.7	3.9974(3)	63.87(1)	162.64(3)
472	0.92	2.6	3.9963(2)	63.82(1)	162.91(4)
540	1.00	2.6	3.9940(3)	63.71(1)	163.48(3)
555	1.05	5.1	3.9934(3)	63.68(2)	163.53(6)
573	1.17	2.7	3.9924(3)	63.63(1)	163.45(3)
573	1.16	2.8	3.9925(3)	63.64(1)	163.48(3)
573	1.22	2.6	3.9924(2)	63.64(1)	163.35(3)
573	1.43	2.6	3.9935(2)	63.69(1)	162.93(3)
573	1.56	2.7	3.9945(2)	63.74(1)	162.65(3)
573	1.72	2.7	3.9954(2)	63.78(1)	162.33(3)
573	1.94	2.5	3.9957(2)	63.80(1)	161.89(3)
573	2.22	2.8	3.9953(2)	63.77(1)	161.34(3)
573	2.40	2.8	3.9946(2)	63.74(1)	161.00(3)
573	2.53	2.7	3.9937(2)	63.70(1)	160.76(3)
573	2.96	2.8	3.9900(2)	63.52(1)	159.96(3)
573	3.20	2.7	3.9874(2)	63.40(1)	159.52(3)
573	3.46	2.9	3.9842(2)	63.25(1)	159.06(3)
573	3.78	2.8	3.9803(2)	63.06(1)	158.50(3)
573	4.13	3.3	3.9753(3)	62.82(1)	157.91(3)
573	4.33	3.2	3.9720(3)	62.67(1)	157.57(3)
573	4.57	3.0	3.9687(3)	62.51(1)	157.18(3)
573	4.80	2.9	3.9649(3)	62.33(1)	156.81(3)
572	4.95	3.0	3.9626(3)	62.22(1)	156.56(3)

Table S2. Unit cell volume per formula unit versus pressure from a Rietveld analysis of the 300 K high-pressure X-ray powder diffraction data assuming that the sample is cubic at all pressures. It is likely not cubic above 5.16 GPa. The underlying data were collected on 13-BMD at GSECARS.

**Pressure (GPa)**	**V/Z (Å^3)**
0.64	63.99(1)
0.66	63.99(1)
0.69	64.0(1)
0.69	64.00(1)
0.71	64.02(1)
0.74	64.02(1)
0.78	64.05(1)
0.83	64.07(1)
0.93	64.13(1)
1.05	64.19(1)
1.14	64.21(1)
1.46	64.24(1)
1.87	64.08(1)
1.93	64.05(1)
2.06	63.99(1)
2.39	63.79(1)
2.83	63.50(1)
3.18	63.28(1)
3.44	63.10(1)
3.72	62.91(1)
4.28	62.47(1)
4.70	62.13(1)
5.16	61.67(1)
5.68	61.12(1)
5.72	60.94(2)
5.84	60.76(2)
6.09	60.38(2)
6.37	59.99(2)
6.77	59.52(2)
7.19	59.00(3)
7.40	58.96(3)
7.65	58.67(3)
7.75	58.60(3)
8.46	57.98(4)
8.57	57.84(4)
8.60	57.84(4)

8.68	57.77(4)
8.76	57.67(4)
8.93	57.56(4)
9.13	57.42(5)
9.32	57.20(5)
9.45	57.09(5)
